# Expression Modulation of Immune Inhibitory Molecules by Small Molecule Inhibitor Drugs in Leukemic Cells of Chronic Lymphocytic Leukemia

**DOI:** 10.5812/ijpr-159353

**Published:** 2025-08-12

**Authors:** Fatemeh Mousavi-Mirkalaei, Saeid Taghiloo, Maryam Alizadeh-Foroutan, Ehsan Zaboli, Mohammad Eslami-Jouybari, Ramin Shekarriz, Leila Mirzakhani, Zohreh Ehsani, Sahar Khosravi, Fatemeh Karimpour, Hossein Asgarian-Omran

**Affiliations:** 1 Department of Immunology, School of Medicine, Mazandaran University of Medical Sciences, Sari, Iran; 2 Gastrointestinal Cancer Research Center, Non-communicable Diseases Institute, Mazandaran University of Medical Sciences, Sari, Iran; 3 Department of Hematology and Oncology, Imam Khomeini Hospital, Mazandaran University of Medical Science, Sari, Iran; 4 Cancer Research Center, Health Research Institute, Babol University of Medical Science, Babol, Iran

**Keywords:** Chronic Lymphocytic Leukemia, Small Molecule Inhibitors, PDL-1, Gal-9, CD200, CD155, HVEM

## Abstract

**Background:**

Targeted therapy with small molecule inhibitors (SMIs) has been considered a highly effective therapeutic strategy for chronic lymphocytic leukemia (CLL). However, there is little information on the detailed mechanisms and association of SMIs with immune evasion mechanisms.

**Objectives:**

This study examined the effects of signaling pathway inhibitors ibrutinib, idelalisib, duvelisib, and venetoclax on the expression of immune checkpoint ligands: Programmed death ligand 1 (PD-L1), galectin-9 (Gal-9), cluster of differentiation (CD)200, CD155, and herpes virus entry mediator (HVEM) in CLL leukemic cells.

**Methods:**

Leukemic cells were isolated from fifteen CLL patients using the magnetic activated cell sorting method, confirmed by flow cytometry, and then cultured and treated with different SMIs for 72 hours. The optimal doses of the applied SMIs for in-vitro treatment were determined by MTT assay. The mRNA expression was measured by real-time PCR assay using β-actin as a housekeeping gene.

**Results:**

The purity of isolated CLL leukemic cells was determined to be more than 97%, as confirmed by dual-color flow cytometry. Based on the IC_50_ results obtained from the MTT assay, the optimal doses of 5, 15.03, 1.07 μM, and 0.5 nM were determined for ibrutinib, idelalisib, duvelisib, and venetoclax, respectively. None of the SMI drugs showed changes in PD-L1 expression levels compared to the untreated group. Additionally, the level of Gal-9 mRNA expression was slightly decreased in all treated groups. The expression level of CD155 was downregulated only after treatment with venetoclax, and an upregulation was observed in the other treated groups. Finally, ibrutinib and idelalisib indicated a mild non-significant upregulation in HVEM gene expression.

**Conclusions:**

Altogether, the treatment of leukemic cells with different SMIs in this study indicated increased or decreased variations in the expression level of immune checkpoint inhibitory ligands in CLL. Therefore, these mechanisms should be considered for further treatment approaches, especially for combinational strategies.

## 1. Background

Chronic lymphocytic leukemia (CLL) is the most common adult leukemia in Western countries (1, 2). Among leukemia, CLL has one of the most familial manifestations and is characterized by the accumulation of cluster of differentiation (CD)19^+^/CD5^+^/CD23^+^ B cells in peripheral blood, bone marrow, and lymphoid organs (2-4). Rai and Binet staging systems are two common CLL classification systems based on the clinical and hematological characteristics of patients (5). Asymptomatic CLL patients at the early stage of the disease do not require treatment and are only monitored unless they show evidence of rapid disease progression (6). The combination chemo-immunotherapy with fludarabine/pentostatin, cyclophosphamide, and rituximab (PCR/FCR) is the current standard of care frontline therapy, which is associated with disease recurrence, toxicity, and side effects (7, 8). Therefore, finding alternative therapies, including targeting signaling pathways, metabolic pathways, or cytokines, can be promising (9). In general, CLL is associated with defects in the immune system.

The crosslinking between leukemic cells with bystander immune and non-immune cells leads to an upregulation in inhibitory immune checkpoint pathways, including programmed death 1/programmed death ligand 1 (PD-1/PD-L1), galectin-9/T cell immunoglobulin and mucin domain-containing protein 3 (Gal-9/Tim-3), CD200/CD200R, lymphocyte activation gene 3/human leukocyte antigen II (LAG-3/HLA-II), T cell immunoreceptor with Ig and ITIM domains (TIGIT)/CD155, and herpes virus entry mediator/attenuating B and T lymphocyte immune checkpoint/CD160 (HVEM/BTLA/CD160), leading to exhaustion and suppression of the immune system cells (10-13).

Among the different types of signaling pathways that exist for B cells, including Wnt, Toll-like receptors (TLRs), and B cell receptors (BCRs) signaling pathways, BCR signaling is the main pathway in CLL cells. It is capable of activating downstream signaling pathways, including Bruton tyrosine kinase (BTK), phosphoinositide 3-kinase/protein kinase B/mammalian target of rapamycin (PI3K/AKT/mTOR), mitogen-activated protein kinases/extracellular signal-regulated kinase (MAPK/ERK), and NF-κB activation, which lead to the proliferation and survival of leukemic cells (4, 14). Besides these molecular pathways, the anti-apoptotic B-cell lymphoma 2 (BCL-2) protein is upregulated in CLL patients, increasing the lifespan of leukemic cells. The BCR binding to its specific antigen leads to the activation of the PI3K signaling pathway (15). Two isotypes of the catalytic subunit of PI3K, including γ and δ isoforms, are limited to leukocytes and hematopoietic cells (16, 17). Activation of PI3K leads to the activation of the AKT pathway and cell processes (18).

Regarding the discrepancies of signaling pathways in tumor cells, signaling pathways targeted therapy by small molecule inhibitors (SMIs) is one of the current new approaches for cancer treatment (19, 20). The SMIs are highly selective and have the ability to bind to a wide range of extracellular and intracellular targets due to their small size (19, 20). In addition, these molecules are associated with lower dosages, longer patient survival, and reduced off-target inhibition, which makes these drugs superior to common CLL treatments.

## 2. Objectives

Thus, considering the association between active signaling pathways in the immune evasion mechanisms and the suppression of the immune system by CLL leukemic cells, this study was performed to evaluate the possible mechanisms of the approved SMI drugs with the expression of immune checkpoint ligands in CLL patients.

## 3. Methods

### 3.1. Selection of Patients and Sample Collection

This in vitro study was conducted on peripheral blood samples isolated from fifteen patients with CLL (Table 1), who were referred to Imam Khomeini Hospital affiliated with Mazandaran University of Medical Sciences. The disease was determined based on clinical evaluations by the attending physician, cell counting, morphological examination in the peripheral blood smear, and immunophenotypic analysis by flow cytometry, based on the WHO criteria. The inclusion criteria were newly diagnosed CLL patients who had not received any immunosuppressive or chemotherapy drugs. Written informed consent was obtained from all participants in accordance with the Declaration of Helsinki, and the study was approved by the Ethical Committee of Mazandaran University of Medical Sciences.

**Table 1.** Demographic Table of Studied Chronic Lymphocytic Leukemia Patients

Sample	Sex	Age	WBC/μL	PLT/μL	Hb g/dL	CD5%	CD19%	CD5/CD19%	CD23%	Rai Stage
**1**	Male	55	57 × 10^3^	201 × 10^3^	13.3	98	97	97	89	II
**2**	Male	69	11.6 × 10^3^	223 × 10^3^	16	97	96	95	92	0
**3**	Male	82	34 × 10^3^	330 × 10^3^	13.5	NA	92	91	90	0
**4**	Male	57	150 × 10^3^	116 × 10^3^	11.6	99	96	94	82	NA
**5**	Female	71	12.6 × 10^3^	310 × 10^3^	11.7	92	70	70	67	0
**6**	Male	79	206 × 10^3^	NA	NA	99	98	97	86	NA
**7**	Male	84	39.4 × 10^3^	125 × 10^3^	11.3	80	85	76	83	0
**8**	Female	62	186 × 10^3^	NA	NA	93	90	87	27	NA
**9**	Male	76	98.6 × 10^3^	202 × 10^3^	11.5	95	75	75	60	0
**10**	Female	78	21 × 10^3^	138 × 10^3^	12	94	95	71	35	0
**11**	Female	61	75 × 10^3^	252 × 10^3^	12	NA	85	85	74	0
**12**	Male	80	31 × 10^3^	NA	NA	95	86	85	87	NA
**13**	Male	50	21 × 10^3^	158 × 10^3^	14.5	92	83	79	73	II
**14**	Female	61	9.9 × 10^3^	60 × 10^3^	11.3	88	93	88	80	NA
**15**	Male	69	14.5 × 10^3^	162 × 10^3^	11.3	NA	75	73	NA	NA

Abbreviations: WBC, white blood cell; PLT, platelet; Hb, hemoglobin; CD, cluster of differentiation; NA, not available.

### 3.2. Isolation of Chronic Lymphocytic Leukemia Leukemic Cells

Initially, 4 - 5 mL of heparinized peripheral blood samples were collected from all CLL patients, and peripheral blood mononuclear cells (PBMCs) were separated using gradient centrifugation (Sigma-Aldrich, USA) in Ficoll Histopaque (Biowest, USA) solution. Isolation of CD19^+^ B-cells was performed by the magnetic-activated cell sorting (MACS) positive selection method using an anti-CD19 microbead antibody according to the protocol of Miltenyi Company (Miltenyi Biotec, Germany). Briefly, isolated PBMCs were washed with MACS buffer (0.15M PBS with 2 mM EDTA and 0.5% BSA) and passed through the LS column to separate the cells bound to the anti-CD19 antibody. To check the purity of isolated CD19^+^ B-cells by flow cytometry, double color staining with CD3-PE and CD5-FITC antibodies (Biolegend, USA) was performed. Isotype-matched control antibodies (Biolegend, USA) were also applied as a negative control in this method.

### 3.3. IC_50_ Determination of Small Molecule Inhibitors in Chronic Lymphocytic Leukemia Leukemic Cells

In this study, the appropriate IC_50_ of each SMI drug was determined on isolated CLL leukemic cells. Four SMIs, including ibrutinib (BTK inhibitor), idelalisib (PI3K δ inhibitor), duvelisib (PI3K δ/γ inhibitor), and venetoclax (BCL2 inhibitor), were applied in this study (Cayman, United States). The MTT assay was used for IC_50_ determination of all SMIs on the CD19^+^ leukemic cells. To determine the optimal number of CLL cells in this study, different numbers of leukemic cells from 25 × 10^3^ to 4 × 10^5^ were cultured in each well of a 96-well plate (SPL, South Korea) in duplicate. Then, the MTT assay was performed, and the best Optical density (OD) was considered according to the cell viability after 72 hours as the optimal number. After that, 3 × 10^5^ CLL cells were seeded in each well of a 96-well culture plate in 200 μL of complete RPMI 1640 medium containing 2 mM glutamine, 10% heat-inactivated fetal bovine serum (Biowest, USA), 100 IU/mL penicillin, and 100 μg/mL streptomycin (Biowest, USA). The cells were treated with different concentrations of the applied SMI drugs, including 0.18 - 12 μM for ibrutinib, 0.62 - 40 μM for idelalisib, 0.12 - 8 μM for duvelisib, and 0.02 - 5 nM for venetoclax, and then incubated for 72 hours in a 37°C incubator containing 5% CO_2_ (Binder, Germany).

After incubation, each well was filled with 20 μL of 5 mg/mL freshly prepared MTT solution (Sigma-Aldrich, USA) and incubated at 37°C for 4 hours. The plates were then centrifuged for 10 minutes at 4°C and 850 g. After centrifugation, the supernatant was carefully removed, and 150 μL of DMSO was added to each well. The plate was shaken in the dark at room temperature for 40 minutes to dissolve all the formazan crystals. The OD values were measured at 570 nm and 720 nm using an ELISA plate reader (Synergy H1 BioTek, USA). In this study, the untreated CLL cells were considered the control group. For IC_50_ determination, incubation was performed at 24, 48, and 72 hours to select the best incubation time. All tests were performed in duplicate.

### 3.4. Treatment of Chronic Lymphocytic Leukemia Leukemia Cells with Optimal Concentrations of Ibrutinib, Idelalisib, Duvelisib, and Venetoclax

A total of 3 × 10^6^ CLL leukemic cells were treated with the optimal dosage of the applied SMI drugs and cultured in each well of a 6-well culture plate (SPL, South Korea) in 3 mL of complete medium. The cells were incubated for 72 hours in a 37°C incubator containing 5% CO_2_. Untreated leukemic cells were considered the control group in this experiment. All tests were performed in duplicate. After incubation, all cells were harvested and used for RNA extraction.

### 3.5. RNA Extraction and Complementary DNA Synthesis

To extract the total RNA from cultured cells, the RNeasy kit (Denazist, Iran) was used. First, the cell suspensions following 72 hours of culture in 6-well plates were collected in 1.5 mL microtubes and centrifuged for 5 minutes at 4°C at 2500 g. The supernatant was then removed, and the RNA extraction process was performed according to the kit protocol. The quality of the extracted RNA was assessed quantitatively and qualitatively using a nano-spectrophotometer (WPA, England) and gel electrophoresis (Bio-Rad, UK), respectively.

Synthesis of complementary DNA (cDNA) was done using a cDNA synthesis kit (Yektatajhiz Azma, Iran) in a 13.5 µL volume containing 12.5 μL of total RNA and 1 μL random hexamer primer. The samples were then placed in the Authorized Thermal Cycler for 5 minutes at a temperature of 70°C. During this period, n + 1 (n was considered as a sample) reaction (including 4 microliters of 5X first standard buffer, 1 microliter of dNTP (10 mM each), RNasin (40 U/microliter), and 1 µL of enzyme MMLV) was prepared, and 6.5 µL was added to the 0.2 microtube containing the sample. It was again placed in the thermal cycler for 60 minutes at 37°C and at the end step for 5 minutes at +70°C. The samples were frozen at a temperature of -70°C until the real-time PCR test was performed.

### 3.6. Semi-quantitative Real-time PCR

To investigate the mRNA expression of PD-L1, Gal-9, CD155, HVEM, and CD200 genes in leukemic cells, the real-time PCR technique was performed using SYBER Green/ROX qPCR Master Mix (Amplicon, Denmark) reagent on an ABI step-one real-time PCR platform (ABI system, USA) with specific primers (Table 2). SYBR Green/ROX reagent was applied to the Real-time PCR reactions. The hot start of the PCR reactions was at 95°C for 10 minutes as initial denaturation, followed by 45 cycles at 94°C for 30 seconds, 60°C (all genes) for 30 seconds, and extension at 72°C for 30 seconds. The PCR amplicon sizes were 174 bp, 159 bp, 118 bp, 124 bp, 147 bp, and 126 bp for β-actin, PD-L1, Gal-9, CD155, CD200, and HVEM, respectively. Each run was completed with a melting curve analysis using Linreg software to confirm the specificity of the amplification curves and the absence of primer dimers. The relative mRNA levels were determined by normalizing the target gene’s fluorescence data to the housekeeping gene β-actin (2^-∆∆Ct^).

**Table 2.** Primers Sequences and Features

Gene	Primers Sequence	Amplicon Size (bp)	Tm (°C)
**PD-L1**	F: CTATGGTGGTGCCGACTACAA; R: CTGCTTGTCCAGATGACTTCG	159	65.6
**Gal-9**	F: TTTCTGGGACTATTCAAGGAG; R: GAAGTGGAAGGCAATGTCA	137	61.4
**CD200**	F: GTCTGTTACCAGCATCCT; R: CTTAGCAATAGCGGAACTG	147	60
**CD155**	F: GGACGGCAAGAATGTGAC; R: CCAGTTGTTATCATAGCCAGAG	124	62.5
**HVEM**	F: TGCTGTATCTCACCTTCC; R: CCTCCTTCACACGATAACC	126	60.6
**β-actin**	F: CCTTCCTGGGCATGGAGTCCT; R: TGGGTGCCAGGGCAGTGAT	174	59

Abbreviations: PD-L1, programmed death ligand 1; bp, base pair; Tm, melting temperature; Gal-9, galectin-9; HVEM, herpes virus entry mediator.

### 3.7. Statistical Analysis

Statistical analysis of data was performed using GraphPad Prism 8.0 (GraphPad Software, USA). The statistical methods used in this project include descriptive methods for the preliminary investigation of independent and dependent variables and analytical methods. Quantitative data are expressed as mean ± SEM. During the data analysis (based on the Kolmogorov-Smirnov and Shapiro-Wilk tests), appropriate analytical tests were used for data with a normal distribution. P-values < 0.05 were considered statistically significant.

## 4. Results

### 4.1. Isolation of CD19^+^ B-Cells and IC_50_ Determination of Ibrutinib, Idelalisib, Duvelisib, and Venetoclax

**Figure 1.**
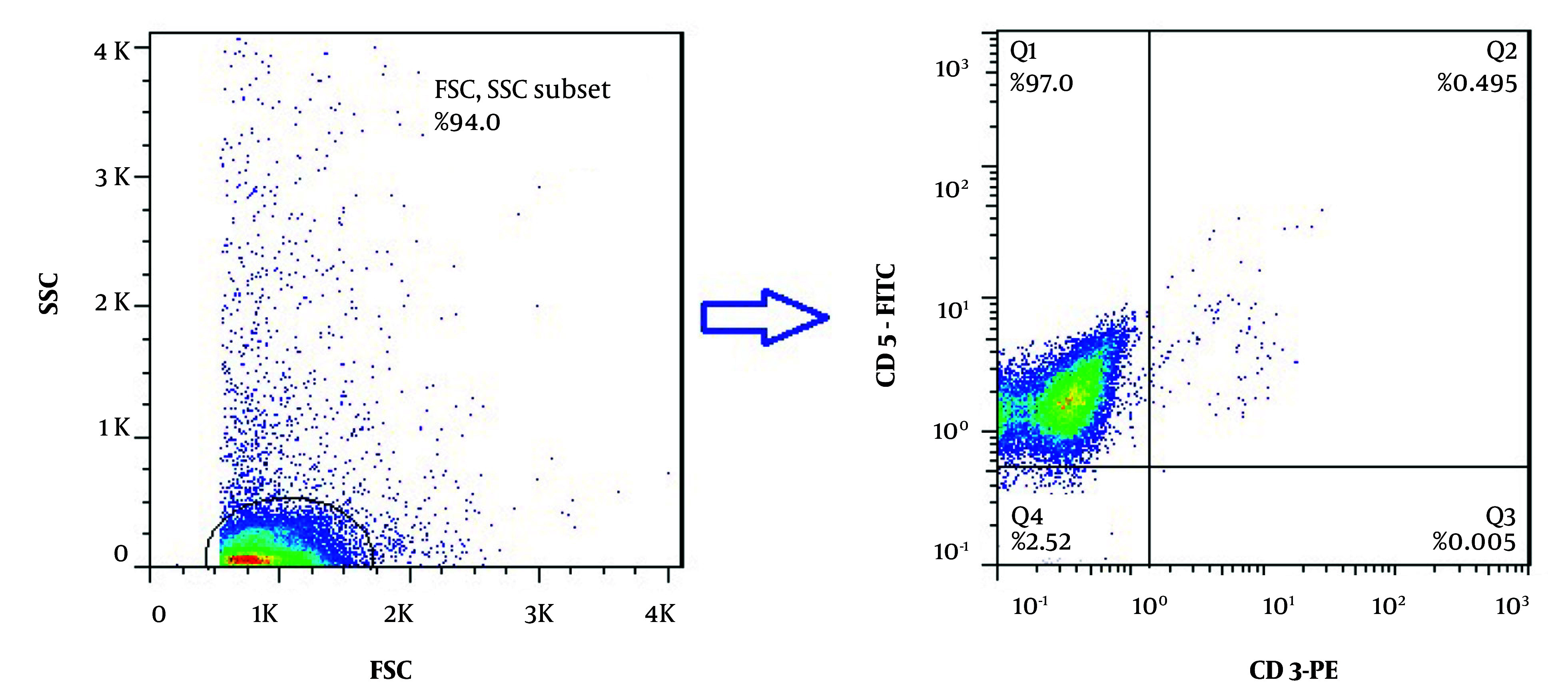
Purity analysis of isolated chronic lymphocytic leukemia (CLL) leukemic cells by flow cytometry; two-color flow cytometry technique using anti-CD5-FITC and anti-CD3-PE antibodies was used to determination the purity of CLL cells isolated by magnetic activated cell sorting method. The purity of isolated leukemic cells (CD5^+^/CD3^-^) was determined to be more than 97% (representative data for a CLL patient is shown).

**Figure 2.**
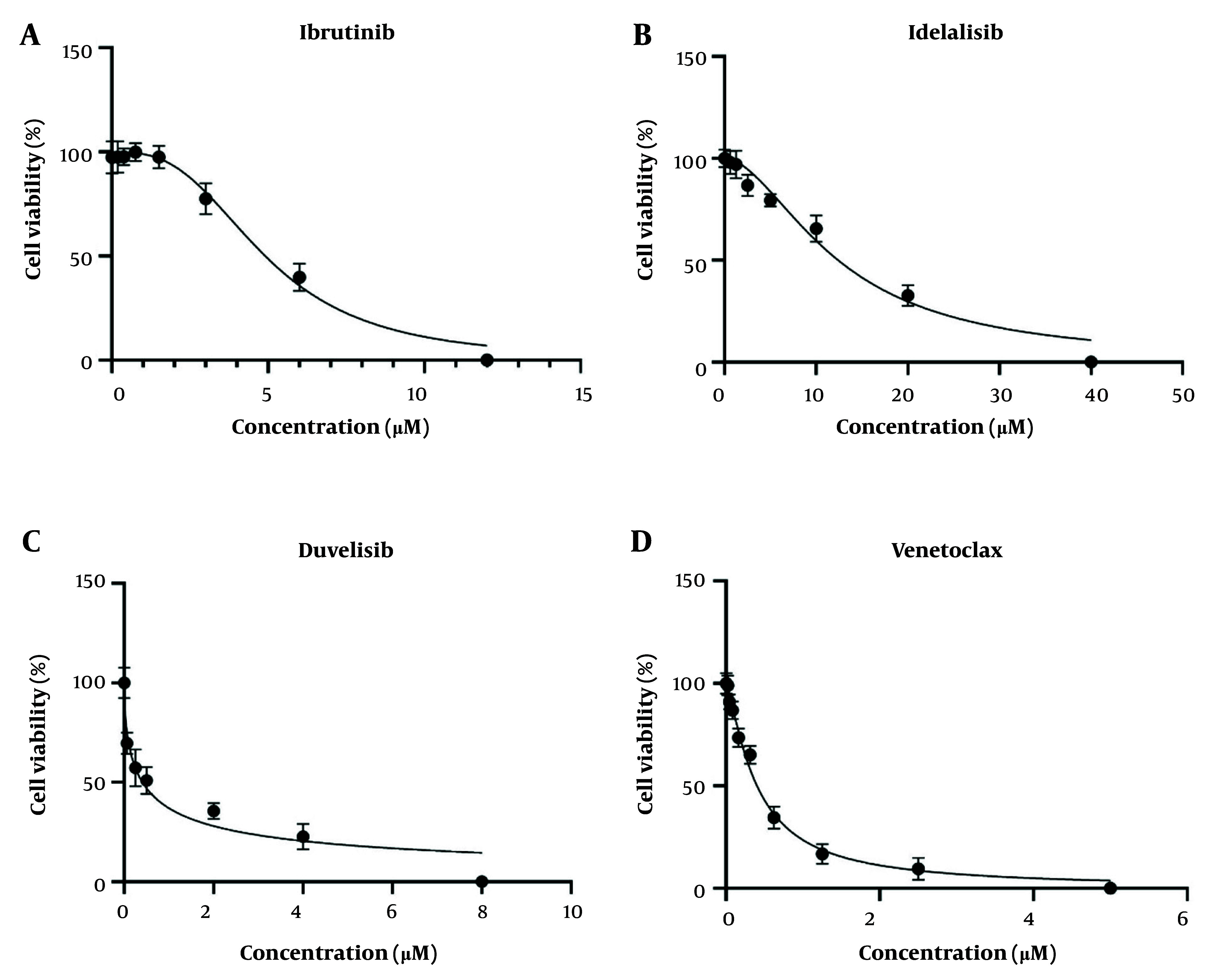
Half maximal inhibitory concentration (IC_50_) values of ibrutinib, idelalisib, duvelisib and venetoclax on chronic lymphocytic leukemia (CLL) leukemic cells; CLL leukemic cells (3 × 10^5^ cells/well) were plated into 96-well culture plates and treated with increasing concentrations of ibrutinib A, idelalisib; B, duvelisib; C, and venetoclax; D, for 72 h (the data are shown as the mean ± SEM; untreated cells were used as control group; representative data for a CLL patient is shown).

Following the isolation of CD19^+^ B-cells by the MACS method, two-color flow cytometric analysis with anti-CD3-PE and anti-CD5-FITC monoclonal antibodies was performed to check the purity of the positively isolated cells. The purity of isolated cells was more than 97%, as represented in Figure 1. The MTT assay was then applied to determine the IC_50_ values of ibrutinib, idelalisib, duvelisib, and venetoclax on leukemic cells isolated from CLL patients following 72 hours of exposure to increasing concentrations of all SMI drugs. Based on the IC_50_ results obtained from the MTT assay, leukemic cell proliferation indicated dose-dependent inhibition in comparison to the untreated group. The optimal IC_50_ doses of ibrutinib, idelalisib, duvelisib, and venetoclax were observed to be 5 µM (CI: 4.47 - 5.57 µM), 15.03 µM (CI: 12.24 - 18.37 µM), 1.07 µM (CI: 0.53 - 1.62 µM), and 0.5 nM (CI: 0.43 - 0.57 nM), respectively (Figure 2). 

### 4.2. Investigation of Cell Viability Following Treatment of Chronic Lymphocytic Leukemia Leukemic Cells with Ibrutinib, Idelalisib, Duvelisib, and Venetoclax

**Figure 3.**
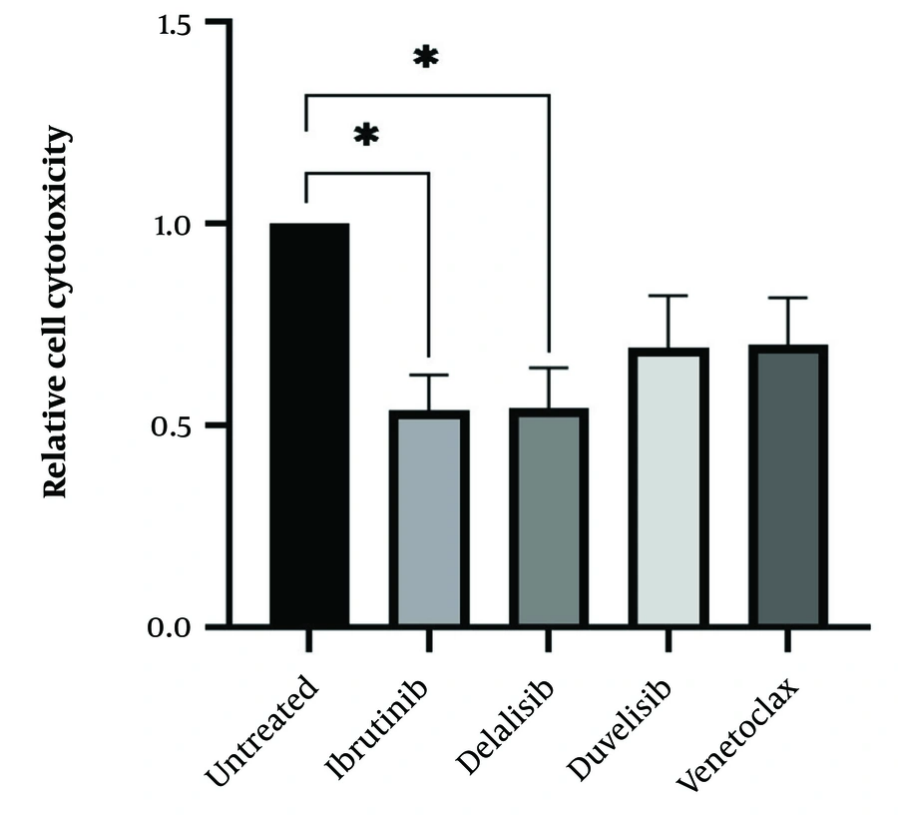
Cytotoxicity of chronic lymphocytic leukemia (CLL) leukemic cells following treatment with small molecule inhibitor (SMI) drugs; isolated leukemic cells from all 15 CLL patients were treated with IC_50_ value of all SMIs to determine the drug cytotoxicity. The CLL leukemic cells showed a significant decrease in cell viability following treatment with ibrutinib and idelalisib (*P < 0.05) and a relative reduction following treatment with duvelisib and venetoclax (the results were reported as mean + SEM; P < 0.05).

After determining the IC_50_ values of all drugs, isolated leukemic cells from all 15 CLL patients were treated with the IC_50_ value of all SMIs to determine drug cytotoxicity. Following 72 hours of culture and treatment of leukemic cells with ibrutinib, idelalisib, duvelisib, and venetoclax, all treated cells indicated a decrease in cell viability compared to the control untreated group. As expected, the results showed a dramatic decrease in the viability of CLL cells following treatment with all SMI drugs, but the statistical analysis showed significance for ibrutinib and idelalisib, as represented in Figure 3. 

### 4.3. Expression Profiles of Immune Checkpoint Molecules following Exposure of Chronic Lymphocytic Leukemia Leukemic Cells to Bruton Tyrosine Kinase, Phosphoinositide 3-Kinase, and BCL2 Inhibitors

**Figure 4.**
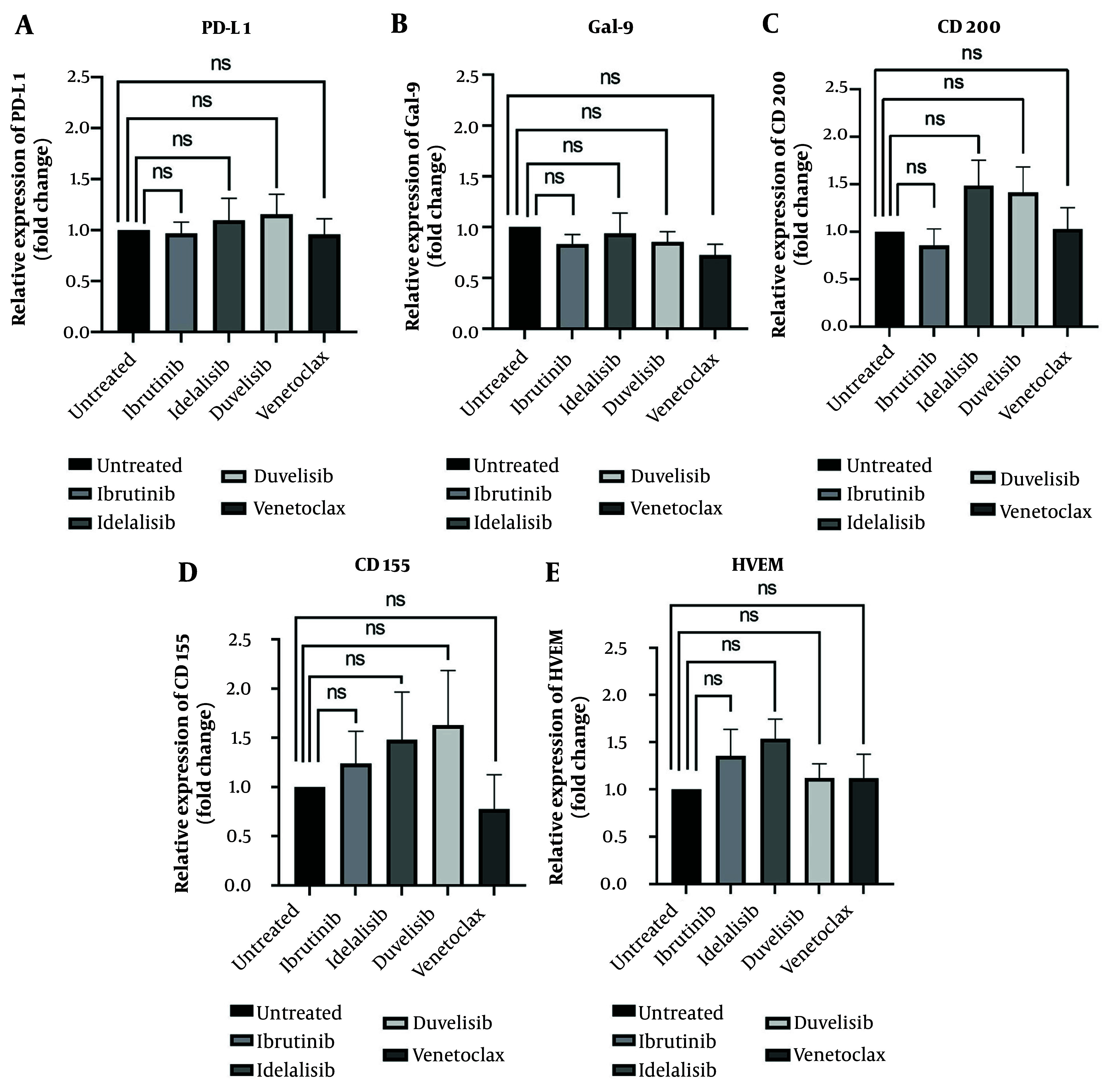
Programmed death ligand 1 (PD-L1), galectin-9 (Gal-9), CD200, CD155 and herpes virus entry mediator (HVEM) mRNA expression in chronic lymphocytic leukemia (CLL) leukemic cells following treatment with ibrutinib, idelalisib, duvelisib and venetoclax; figures A, B, C, D and E indicate fold changes in PD-L1, Gal-9, CD200, CD155, and HVEM molecules following CLL cells treatment with ibrutinib, idelalisib, duvelisib, and venetoclax, respectively. The results of mRNA expression were performed by real-time RT-PCR test and β-actin was used as a housekeeping control (the results were reported as mean + SEM; P < 0.05).

To better understand the molecular events underlying immune evasion in CLL leukemic cells and the effects of BTK, PI3K, and BCL2 inhibitors on these mechanisms, the mRNA expression profile of immune checkpoint ligands PD-L1, Gal-9, CD200, CD155, and HVEM were evaluated in leukemic cells following treatment with ibrutinib, idelalisib, duvelisib, and venetoclax. The obtained data demonstrated that none of the SMI drugs showed changes in PD-L1 gene expression levels compared to the untreated group (Figure 4A). Additionally, the level of Gal-9 mRNA expression was slightly decreased in all treated groups, but the statistical analysis was not significant (Figure 4B). Regarding CD200, the mRNA expression level of this molecule indicated a non-significant decrease in CLL cells following treatment with ibrutinib and also showed a non-significant increase following inhibition of the PI3K δ and dual PI3K δ/γ pathways by idelalisib and duvelisib (Figure 4C). Interestingly, the expression level of CD155 was downregulated only after treatment with venetoclax, whereas in the other treatment groups, an upregulation in the mRNA expression level of CD155 was observed (Figure 4D). Finally, ibrutinib and idelalisib indicated a mild non-significant upregulation in HVEM gene expression, but this was not observed for duvelisib and venetoclax (Figure 4E). Altogether, although slight differences were observed in the mRNA expression of immune checkpoint molecules following treatment with all applied SMIs in CLL leukemic cells, all differences were non-significant in comparison to the control group.

## 5. Discussion

Traditional treatments for cancer therapy include chemotherapy, radiotherapy, and surgery. These therapeutic methods are used for many malignancies, but due to their destructive effects on both cancer and healthy cells, several research efforts have been carried out to apply specific targeted strategies to avoid the harmful effects of these methods (21). Therefore, two types of treatment methods that are more specific than the common treatments, including SMIs and monoclonal antibodies, have gained attention and expanded more than before, as they can selectively target cancer cells (22). Due to the much lower relative mass of SMIs compared to monoclonal antibodies, SMIs are able to penetrate the cell membrane and target specific intracellular molecules (23).

Regarding the available and approved SMI drugs for the treatment of CLL, their exact mechanisms and effects on immune checkpoint molecules are not fully understood. The current study examined the effects of four SMIs, including ibrutinib, idelalisib, duvelisib, and venetoclax, on the expression profile of immune checkpoint ligands in CLL leukemic cells.

It has been shown in CLL that ibrutinib directly affects leukemic cells and reduces their immunosuppressive function, as well as modulates the tumor microenvironment and restores the physiological functions of the immune system by increasing the survival of T-cell mediated immunity (24). Additionally, it has been reported that ibrutinib leads to a decrease in the frequency of peripheral blood T-cells expressing immunosuppressive ligands such as PD-L1 (25), CD200, and BTLA (26) in CLL cells, as well as decreased production of IL-10 (25, 26). These results are partially aligned with our finding that the expression level of the CD200 inhibitory ligand decreases following the treatment of CLL leukemic cells with ibrutinib. However, contrary to the results from the above study (25), PD-L1 did not show any differences compared to the untreated group. It should be noted that these discrepancies may be due to different methodological approaches. Kondo et al. measured the frequency of PD-1 expressing T-cells and PD-L1 expressing leukemic cells in CLL patients following treatment with ibrutinib (25). Long et al. also evaluated the frequency of PD-1 expressing T-cells after monotherapy with ibrutinib (26). In our study, isolated CLL leukemic cells were cultured and directly treated with ibrutinib, and then the expression of checkpoint molecules was investigated.

It was also reported in acute lymphoblastic leukemia that ibrutinib has no effects on the expression of the PD-L1 molecule (27), but in the MCF-7 breast cancer cell line, it could reduce the expression of PD-L1 (28). However, ibrutinib led to a non-significant downregulation of Gal-9 and an upregulation of HVEM and CD155 gene expression in leukemic cells following in-vitro treatment. Previous findings emphasize that the expression of CD155 is regulated by transcription factors AP-2, nuclear respiratory factor, and sonic hedgehog, and the Rad-MEK-ERK signaling cascade is responsible for activating the mentioned transcriptional factors (29, 30).

In the present study, it has been shown that idelalisib, by inhibiting the PI3K δ signaling pathway in CLL cells, led to a non-significant decrease in the level of Gal-9 gene expression, and also a non-significant increase in the expression of CD200, CD155, and HVEM. Additionally, based on the results of another study that investigated the effects of PI3K/AKT/mTOR signaling pathway inhibitors on the expression of inhibitory immune checkpoints, including PD-L1, CD155, and Gal-9 in the AML cell line (HL-60), it was reported that blocking these signaling pathways with idelalisib and everolimus leads to a downregulation in the expression of PD-L1 and Gal-9. It was also demonstrated that combination treatment of the AML cells could further reduce the expression of these inhibitory ligands (31). Similar results were also reported in in vitro and mouse models of AML following inhibition of the PI3K/mTOR pathway (32, 33).

In another research conducted on CLL, it was concluded that the STAT3 transcription factor, which is activated by BTK molecules, upregulates PD-L1 expression (25). However, PD-L1 expression in lung adenocarcinoma is controlled by the signaling pathway originating from MAPK (34). These different results regarding PD-L1 expression in various malignancies are due to differences in the derivation of tumor cells from different cell origins. It has also been shown that duvelisib, as a dual oral inhibitor of PI3K δ and PI3K γ isoforms, leads to an upregulation in BCL-2 protein during the treatment of CLL patients (35).

As previously reported, and based on treatment with ibrutinib and idelalisib, the upregulation of BCL-2 in concert with BH3-only pro-apoptotic proteins raised the possibility that duvelisib treatment may not induce significant apoptosis by itself. However, it may increase sensitivity to BCL-2 inhibition by venetoclax when CLL cells were previously exposed to idelalisib (36).

Mantle cell lymphoma (MCL) is a B-cell malignancy associated with high expression of the inhibitory ligand PD-L1 and variable expression of CD200 on the surface of malignant cells. Induction of PD-L1 in malignant MCL cells and simultaneous treatment with ibrutinib or duvelisib leads to a downregulation in this inhibitory ligand, indicating the role of BTK and PI3K pathways in the expression of PD-L1 (37). Following duvelisib treatment of CLL cells in this study, the results showed a non-significant downregulation in Gal-9 gene expression and a relative upregulation in the expression levels of CD200 and CD155 ligands in leukemic cells, but no changes in the expression levels of PD-L1 and HVEM were observed.

Venetoclax is known as a promising SMI that targets the BCL-2 protein, whose overexpression is one of the molecular hallmarks of CLL. This drug is effective both in first-line treatment and in relapsed/resistant patients and is associated with complete recovery and long progression-free survival in these patients (38). Targeting the anti-apoptotic protein BCL-2 by venetoclax in this study shows a downregulation in Gal-9 and CD155 gene expression in CLL leukemic cells, which is promising. However, the results do not show differences in the expression levels of PD-L1 and HVEM.

### 5.1. Conclusions

Collectively, in vitro treatment of isolated CLL leukemic cells with ibrutinib, idelalisib, duvelisib, and venetoclax indicated increased or decreased non-significant variations in the expression levels of immune checkpoint inhibitory ligands, including PD-L1, Gal-9, CD200, CD155, and HVEM. Thus, it can be concluded that the applied SMIs are not only effective in preventing the proliferation and expansion of tumor cells but also play a role in mediating immune system suppression and exhaustion processes. These mechanisms could be considered for further treatment approaches in CLL and other malignancies, especially for combinational strategies. However, more studies are required to expand these data and determine the exact detailed mechanisms.

## Data Availability

The dataset presented in the study is available upon request from the corresponding author during submission or after its publication.
